# Mechanical performance and thermal stability of hardened Portland cement-recycled sludge pastes containing MnFe_2_O_4_ nanoparticles

**DOI:** 10.1038/s41598-023-29093-y

**Published:** 2023-02-04

**Authors:** O. A. Mohamed, S. I. El-dek, S. M. A. El-Gamal

**Affiliations:** 1 Environmental Science and Industrial Development Department, Faculty of Postgraduate Studies for Advanced Sciences (PSAS), Beni-Suef University, Beni-Suef, Egypt; 2 Materials Science and Nanotechnology Department, Faculty of Postgraduate Studies for Advanced Sciences (PSAS), Beni-Suef University, Beni-Suef, Egypt; 3 Chemistry Department, Faculty of Science, Ain Shams University, Cairo, Egypt

**Keywords:** Engineering, Materials science

## Abstract

This study focused on investigating the possibility of using different ratios (5, 10, 15 mass%) of recycled alum sludge (RAS) as partial replacement of ordinary Portland cement (OPC), to contribute to solving the problems encountered by cement production as well as stockpiling of large quantities of water-treated sludge waste. MnFe_2_O_4_ spinel nanoparticles (NMFs) were used to elaborate the mechanical characteristics and durability of different OPC-RAS blends. The outcomes of compressive strength, bulk density, water absorption, and stability against firing tests fastened the suitability of utilization of RAS waste for replacing OPC (maximum limit 10%). The inclusion of different doses of NMFs nanoparticles (0.5, 1 and 2 mass %) within OPC–RAS pastes, motivates the configuration of hardened nanocomposites with improved physico-mechanical characteristics and stability against firing. Composite made from 90% OPC–10% RAS–0.5% NMFs presented the best characteristics and consider the optimal choice for general construction applications. Thermogravimetric analysis (TGA/DTG), X-ray diffraction analysis (XRD), and scanning electron microscope (SEM) techniques. affirmed the positive impact of NMFs particles, as they demonstrated the formation of enormous phases as ilvaite (CFSH), calcium silicate hydrates (CSHs), MnCSH, Nchwaningite [Mn_2_ SiO_3_(OH)_2_ H_2_O], [(Mn, Ca) Mn_4_O_9_⋅3H_2_O], calcium aluminosilicate hydrates (CASH), Glaucochroite [(Ca, Mn)_2_SiO_4_, and calcium ferrite hydrate (CFH). These hydrates boosted the robustness and degradation resistance of the hardened nanocomposites upon firing.

## Introduction

Lately, an enormous leap in the construction field was observed, where the attempts of researchers have become extremely great and focused on how finding convenient alternatives for cement (partially or totally replaced) in concrete^1^. Approximately ~ 5% of global greenhouse gas emissions result from the cement industry (~ 1 ton of CO_2_ is produced during the manufacturing of one ton of Portland cement). Additionally, the cement industry becomes very expensive and highly consumed by energy and natural resources. So, solving the economic and ecological problems of the cement production industry has become extremely urgent^2,3^.

Fortunately, recycling some industrial wastes becomes an essential way to the crucial challenges mitigate their risks in the future. So, usage of these wastes in the building industry sector has several returns that mainly reduce landfill area, cost saving, energy saving, environmental protection where the peril for human health is minimized, and saving resources^4–6^. In previous and recent studies for numerous researchers, plenty of solid byproducts (industrial wastes or agricultural) have been reused in sustainable construction fields. Ceramic waste^7,8^, waste marble dust^9,10^, glass wastes^11^, fly ash^12,13^, brick waste^14^, bagasse ash^15^, rice husk ash^16^ slag (GGBFS)^17^ and silica fume (SF) are famous examples for theses solid byproducts^18^.

The disposal of water treatment sludge (WTS) becomes a serious international problem^19^. Generally, the draining of excessive sludge obtained during water treatment by either unloading it into waterways or discharging it to landfills is deemed a big environmental problem^20^. The special features of water treatment sludge are strongly encouraged its usage in partially replacing clay required for the manufacturing of clinker and other sintered ceramic materials to minimize environmental risks, cost–benefit, and collaboration in sustained building materials production^21^. Several studies evaluated the feasibility of using water treatment sludge (WTS) in supplementary cementitious materials for concrete^22^ and mortar^23^.

Spinel compounds have the formula DT_2_O_4_ (while; D represent divalent cation, like: Ca, Mg, Cu, Ni, Fe, Mn, Co, and Zn while, T represent trivalent metal, like Al, Fe, and Cr) and are technologically substantial materials because of their physical characteristics. The Spinel ferrites possess a crystallographic geometry of [M^2+^-] tetra [Fe^3+^]_octa_ O_4_ where M^2+^ represent divalent ion as Mg^2^, Ni^2+^, Co^2+^, Zn^2+^, Cu^2+^, Fe^2+^ and Mn^2+^^2^.

Recently, nanomaterials are utilized heavily in the construction field where that many researchers reported the affirmative impacts of nanomaterials on the densification performance of hardened cement pastes; as nano SiO_2_, TiO_2_, CNTs, Fe_2_O_3_, ZnO, and Montmorillonite^24–31^ and spinel nanoparticles (NiFe_2_O_4_, ZnFe_2_O_4_, and Cu Fe_2_O_4_) on different aspects of neat OPC and blended OPC hardened pastes^32–34^.

In addition, the use of nanoparticles is one of the effective ways to achieve sustainable development goals (SDGs) in the field of construction, which can engage in reducing pollution from the production and consumption of cement^35^.

In this respect, the unique chemical, and physical features of nanoparticles, compared to conventional materials (i.e., fly ash FA and silica fume SF, …. etc.), such as large surface area and nanoparticle dispersion can reinforce the nature of cementitious materials on both micro and nano scales, causing advanced properties such as; performance and microstructure of cement-based materials^36,37^.

The influences of various types of NP on the durability, mechanical, microstructure, and thermal properties of concrete, mortar, and pastes composites have been studied^38–40^ for example; the magnificent mechanical features, low density, compact cement matrix, biological degradation, and improved cement hydration are abundant benefits of utilizing Nanocellulose (NC), (as low-cost, an environmentally-friendly, and effective material), in cement-based materials^39^. Newly, the positive impact of CNTs for Alkali-silica reaction (ASR) resistance has been achieved through some basic mechanical and chemical mechanisms as follows; enhancing the pore structure, inhibiting cracks, improving the hydration process, and changing pore solution composition^41^.

The influence of the inclusion of Mn-Ferrite nanoparticles on the mechanical strength and toughness of SCC (Self-Compacting Concrete) was investigated. The outcomes indicated that the inclusion of 0.5% MnFe_2_O_4_ spinel NPs, by mass accelerated the hydration process and generated extra quantities of products via its reaction with portlandite generated from silicate phases of cement clinker that intern causes an improvement of the densification characteristics and durability of SCC^42^.

This investigation focused on the utilization of RAS as a partial substituent for OPC to contribute to solving the problems encountered by cement production. As well as stockpiling huge quantities of water-treated sludge waste. Choosing the best replacing ratio of RAS for obtaining blended composite with optimum features (good strength and durability).

Many investigations were published that concerned with investigating the effect of different nanomaterials on the physicochemical and mechanical properties of blended cement. But to our knowledge, there are no published studies that deal with investigating the influence of MnFe_2_O_4_ spinel on the mechanical, durability, and densification performance of OPC pastes replaced with RAS. NMFs are one of the most important magnetic metal oxide nanoparticles with distinguishing chemical and physical properties^43^. This spinel is low cost and easily synthesized through various methods with controllable size and desired morphology. Besides MnFe_2_O_4_ is an efficient candidate for various applications as biomedical, analytical, and storage devices. So, this study focused on investigating the possibility of utilizing this nano spinel for elaborating the durability of various OPC-RAS pastes^44^.

## Materials and experimental tests

### Materials

**Figure 1:**
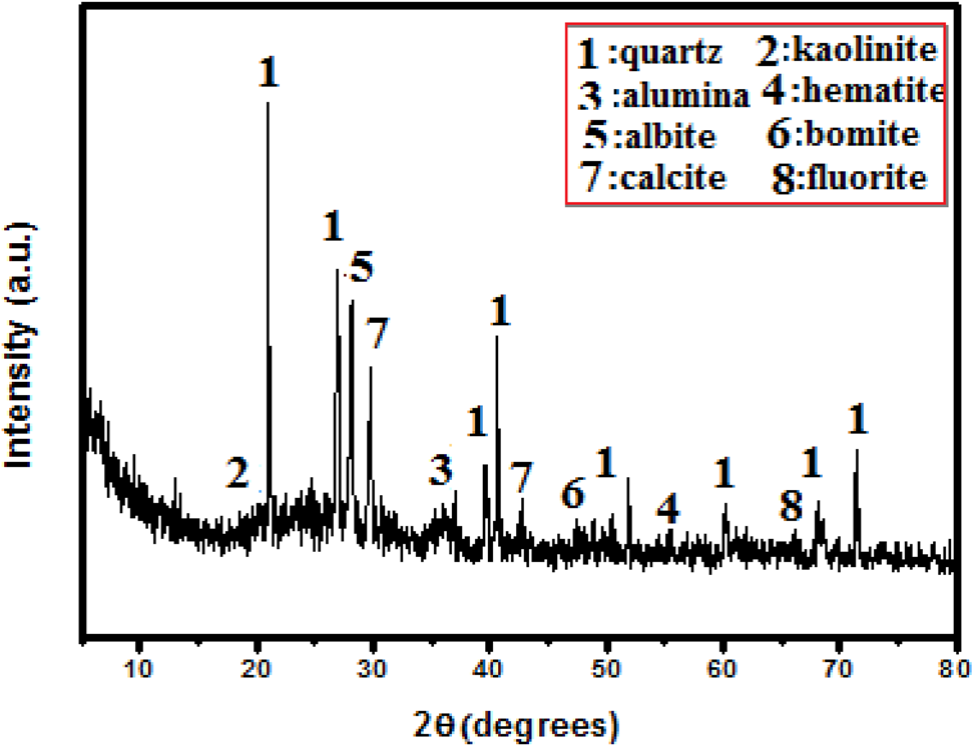
XRD pattern of RAS.

Type I-OPC has Blaine area 349.5 m^2^/kg delivered from El-Sewedy cement group, Al-Ain Al-Sokhna, Suez, Egypt, and water treatment plant sludge (WTPS), delivered from Beni-Suef water treatment plant for purifying drinking water, were used in this work. First WTPS was dried for 1 day at 105 °C. The dried and crushed sludge was thermally activated by firing for 120 min in an electric furnace at 500 °C. The heating rate was 5 °C/min. Finally, the formed sludge (recycled alum sludge, RAS) was left to cool gradually. XRD patterns of RAS are clarified in Fig. 1. The oxides percentages of OPC and the recycled sludge were demonstrated in Table 1a and the mineralogical phase composition of OPC is represented in Table 1b.

**Table 1:** (a) The oxide composition of OPC and recycled alum sludge, (b) mineralogical phase compositions OPC (mass, %).

Oxides	OPC (mass %)	RAS
(a)		
CaO	62.30	6.80
SiO_2_	19.20	62.64
Al_2_O_3_	4.90	13.66
Fe_2_O_3_	3.60	5.00
MgO	2.44	1.50
SO_3_	2.80	0.65
Na_2_O	0.41	0.48
K_2_O	0.63	0.76
Cl^−^	0.09	0.182
L.O.I	3.63	
LSF	–	3.94
(b)		
C_3_S	51.52	
C_2_S	16.26	
C_3_A	6.95	
C_4_AF	10.96	

According to TS 25^45^, The pozzolanic activity of activated alum sludge waste (AAS) as raw material in the production of supplementary cementitious was evaluated through compliance with the chemical and physical requirements of standards applicable to normal pozzolanic material where TS 25 standard suggests that SiO_2_ + Al_2_O_3_ + Fe_2_O_3_ should be at least 70%, SO_3_ < 3.0%, CI^−^ < 0.1%, the compressive strength at the end of 7 days > 4 N mm^−2^ and the reactive silica content of pozzolanas > 25%. So, it can be seen that the results of Table 1a show that all samples comply with the requirements related to the use of activated alum sludge waste (AAS) as supplementary cementitious materials (SCM) with properties equivalent to those of a normal active pozzolanic material^45^.

Modified polycarboxylate-based superplasticizer, SP (Sika Viscocrete 5230 L) supplied from Sika Company, Elobour City, Egypt, was utilized to help in dispersing the spinel nanoparticles in the water used for paste preparation and to attain the desired workability of the formed pastes^27^, some important features of it are reported in Table 2.

**Table 2:** Physical properties of polycarboxylate super plasticizer (SP).

Appearance	Yellow–brown liquid
Solid residue (%)	Approximately 39.9%
pH	7.51–7.53
Specific gravity (g/ml)	Approximately 1.08

**Figure 2:**
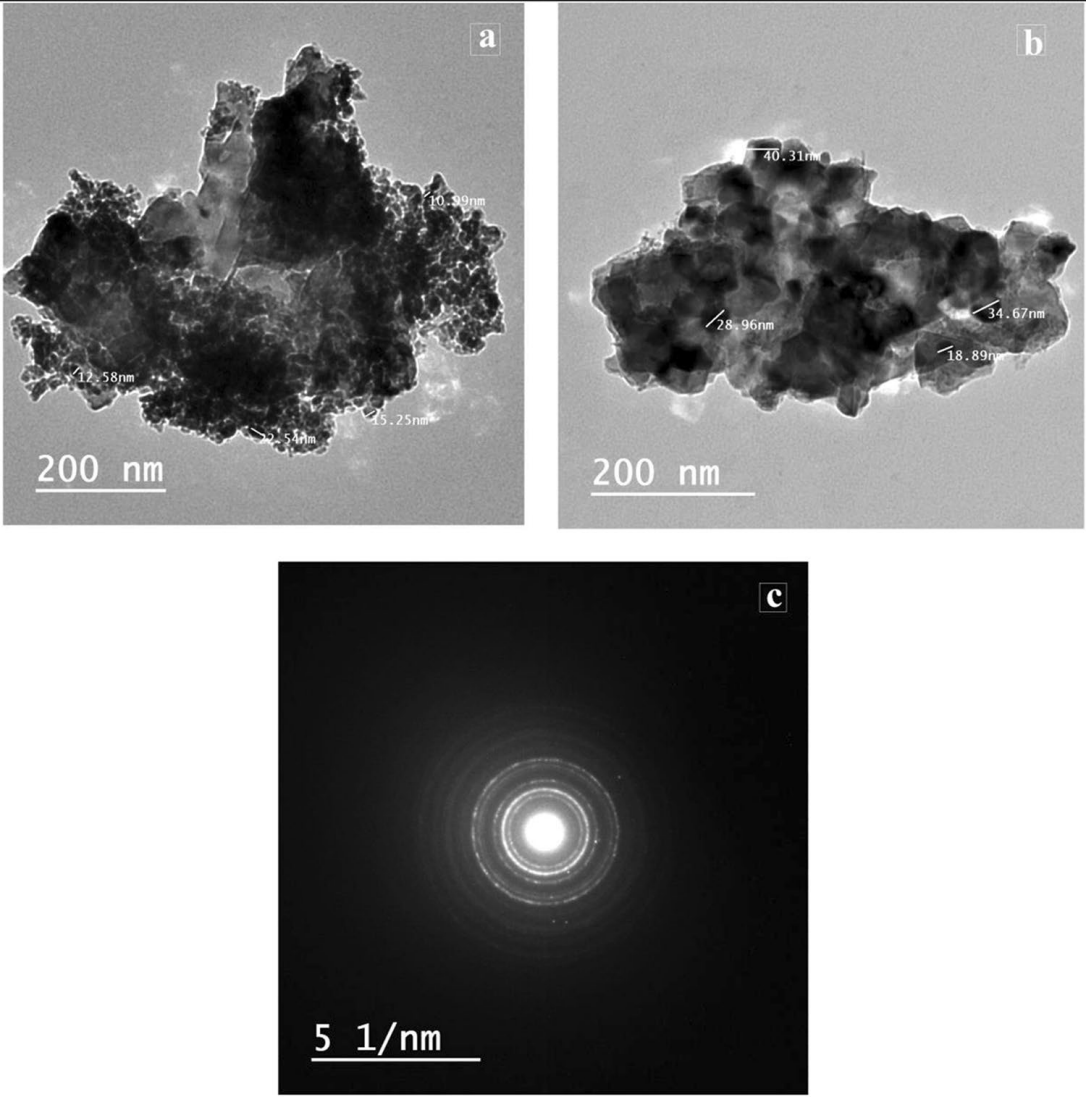
HR-TEM and SAED micrographs of of MnFe_2_O_4_ nanoparticles.

**Figure 3:**
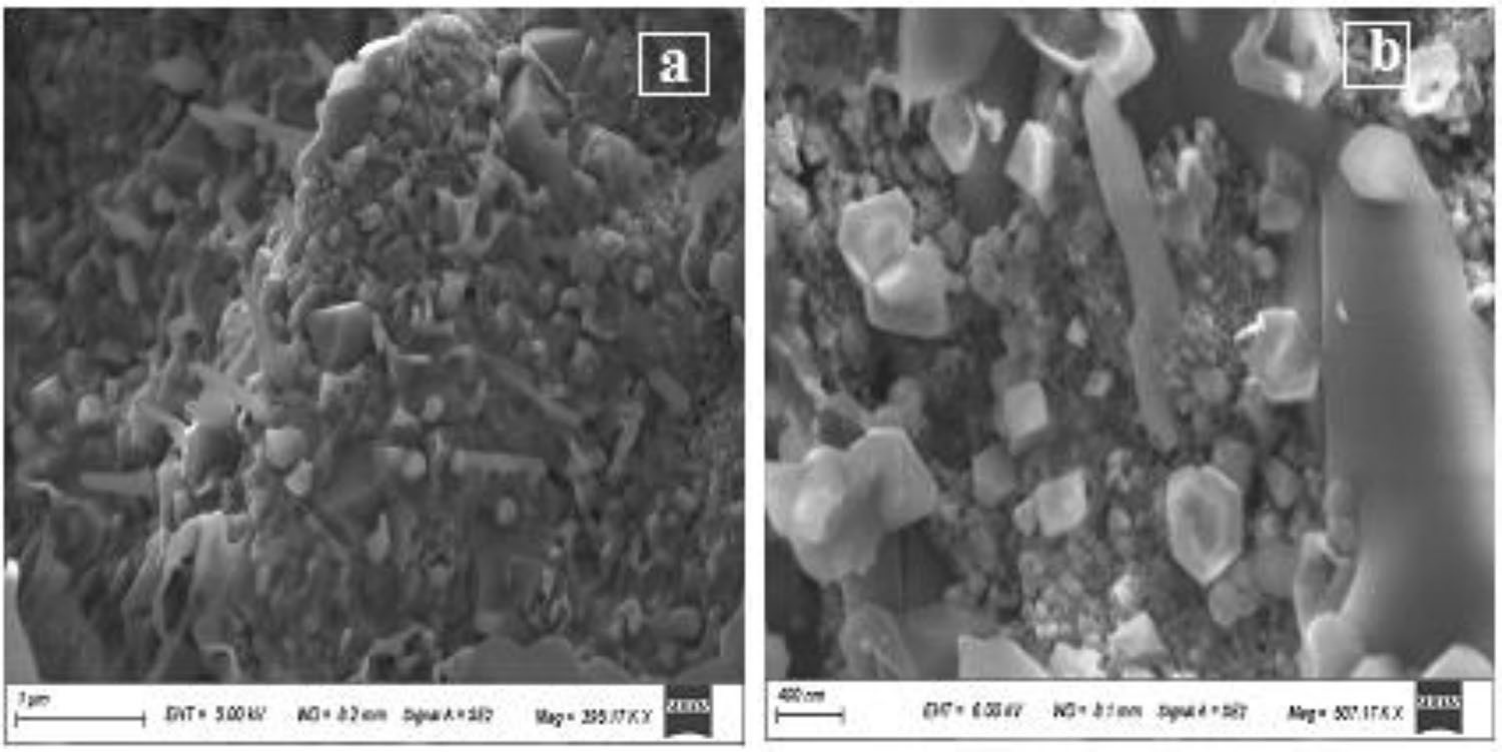
SEM of MnFe_2_O_4_ nanoparticles.

**Figure 4:**
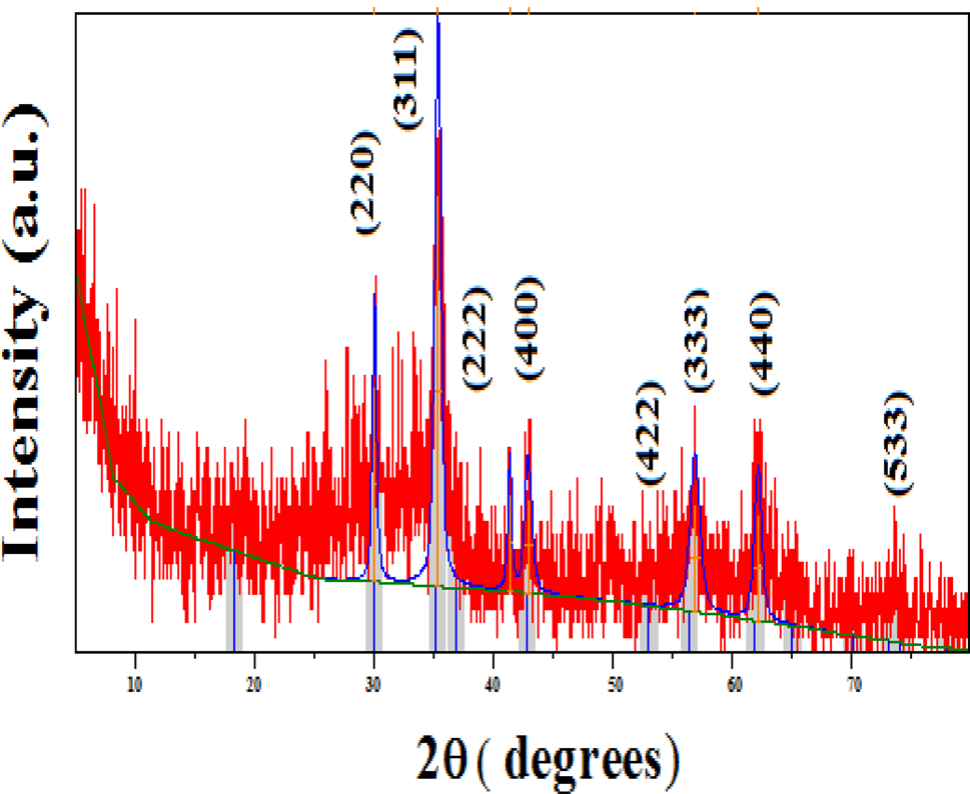
XRD pattern of manganese ferrite nanoparticle (MnFe_2_O_4_).

**Figure 5:**
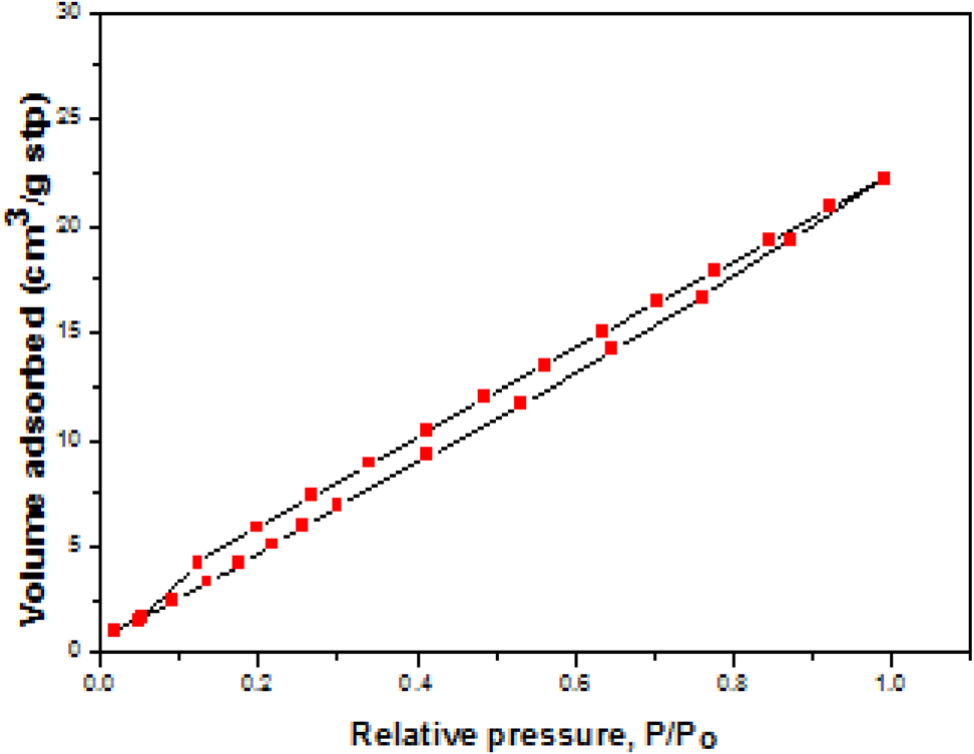
N2 adsorption–desorption isotherms of manganese ferrite spinel nanoparticle.

Mn-Fe_2_O_4_ nano spinel was laboratory synthesized using citrate precursor via a technique reported in previous research^46^. A stoichiometric masses of manganese nitrate {Mn (NO_3_)_2_⋅6H_2_O}, iron nitrate {Fe (NO_3_)_3_⋅9H_2_O} where (Mn:Fe = 1:2) were mixed; citric acid {C_6_H_8_O_7_⋅H_2_O} was inserted by dropwise addition with continuous stirring to form a citrate precursor mixture. To retain a neutral medium (pH = 7), drops of NH_4_OH were inserted into the precursor solution, finally; the precursor mixture was evaporated to get the desired spinel nanocomposite as uniformly colored gray fibers. The prepared Mn-Fe_2_O_4_ spinel nanoparticles (NMFs) have a 30.73 m^2^ g^−1^ surface area and purity > 90%. Lattice parameter *a* (Å) ~ 8.4990, with ~ 5.12 g cm^−3^ x-ray density (D_**χ**_). HR-TEM, SEM, XRD, and N_2_-adsorption/desorption analyses for (NMFs) are demonstrated in Figs. 2, 3, 4, 5, respectively. Table 3 illustrates some features of Mn-Fe_2_O_4_ spinel nanoparticles (NMFs).

**Table 3:** General properties of the synthesized MFs NPs spinel nanoparticles.

Crystallite size (nm)	Room temperature χ_M_ (emu g^−1^) mole	Curie temperature T_C_ (K)	Room temperature dielectric constant (ϵ′) at 100 kHz	Dielectric constant (ϵ′) at 100 kHz–500 K	Room temperature conductivity (Ω^−1^ m^−1^) at 100 kHz	Room temperature dielectric loss (tanδ) at 100 kHz
~ 16	5.76	613	28.46	61.25	1.38 × 10^4^	0.80

### Methodology and experimental techniques

OPC was partially replaced with 5%, 10%, and 15% RAS, by mass. Each dry component was introduced to the ball mill for enough time (~ 6 h) to achieve a homogenous dry mixture. Pastes of different blends were developed using water to a solid ratio (W/S) of 27% from the whole solid mass. To prepare nanocomposites pastes the desired doses of Mn-Fe_2_O_4_ spinel nanoparticles (NMFs) were inserted into the whole water of mixing that containing SP superplasticizer as dispersing reagent (SP amount used was 0.3% by mass of solid). Mn-Fe_2_O_4_ spinel nanoparticles are dispersed in an aqueous solution (i.e., water and SP superplasticizer) before mixing with cement. The whole suspension was sonicated at 25 °C for 60 min to disperse NMFs and prevent their agglomerations. An ultrasonic device (bath machine) (Ultrasonic Homogenizer, LUHS0A12, 650 W, 220 V/50HZ) was used for this step. The samples composition and notations are given in Table 4.

**Table 4:** The percentage composition of the different mixes and their designations.

Mix notation	Mix proportions (mass%)				W/S ratio
	OPC	RAS	(MFs NPs)	Superplasticizer	
A	100	–	–	0.30	0.27
ANMFs0.5	100	–	0.5	0.30	0.27
ANMFs1	100	–	1.0	0.30	0.27
ANMFs2	100	–	2.0	0.30	0.27
B	95	5	–	0.30	0.27
BNMFs0.5	95	5	0.5	0.30	0.27
BNMFs1	95	5	1.0	0.30	0.27
BNMFs2	95	5	2.0	0.30	0.27
C	90	10	–	0.30	0.27
CNMFs0.5	90	10	0.5	0.30	0.27
CNMFs1	90	10	1.0	0.30	0.27
CNMFs2	90	10	2.0	0.30	0.27
D	85	15	–	0.30	0.27
DNMFs0.5	85	15	0.5	0.30	0.27
DNMFs1	85	15	1.0	0.30	0.27
DNMFs2	85	15	2.0	0.30	0.27

#### Experimental test procedure

After the complete mixing of dry powder with the desired quantity of water (or water containing NMFs) the formed pastes were inserted in cubic molds (25 × 25 × 25 mm^3^) and kept for 1 day at ~ 100% RH (relative humidity). After the complete set, the cubic specimens were separated from the mold and cured under tap water at ambient temperature for different intervals till reach 28 days. "Ton-industry (West Germany) machine (60 tons maximum load) was used to perform the test of strength (compressive strength) was used to perform the test of strength (compressive strength) for different samples after 1, 3, 7, and 28 days (the average value of 4 cubes was recorded for each time). To perform XRD, SEM, and TGA/DTG tests, the hydration process was stopped by stirring the grounded samples in acetone and methanol mixture (1:1 by volume) for ~ 45 min and washing the filtrated solid with acetone several times (3–4 times), then it left in the drier at 80 °C overnight. The dried sample is kept in a desiccator (containing silica gel) till performing the other tests^47^. For XRD and TGA/DTG analysis, the particle size used was in the range of 10–50 µm. While for SEM analysis a small piece with ~ 1.5–2 mm thickness from the core of the crushed cube was used.

Important physical properties, like BD (bulk density), (%TP) total porosity, and (WA %) water absorption was measured at defined curing ages^48^, the applied methods are as stated in the following relations were applied:

1 $$ {\text{BD }}\left( {{\text{g cm}}^{{ - {3}}} } \right) = {\text{ W2}}/\left( {{\text{W}} - {\text{ W1}}} \right) $$

2 $$\mathrm{TP }\left(\mathrm{\%}\right)= W-W2/(W-W1) \times 100$$

3 $$ {\text{WA }}\left( \% \right)  = \left[ {\left( {{\text{W }}{-}{\text{ W2}}} \right){\text{/W2}}} \right] \times 100 $$

where W, W1, and W2 are the saturated, suspended, and dried weights; respectively.

For performing the resistance to high temperatures test, cubic molded pastes were cured for 28 days underwater and then dried at 80 °C for 1 day. Then, the cubes were fired at 300, 600, and 800 °C for 180 min. After firing the specimens subjected to cooling in two different ways (fast and slow)^49^, the strength (compressive strength) test was performed on the cooled cubes.

Scanning electron microscopy (SEM), X-ray diffraction (XRD), and thermogravimetric analysis (TGA/DTG) techniques were adopted to identify microstructure, phase presence, and thermal changes for some selected samples.

## Results and discussion

### Mechanical characteristics

**Figure 6:**
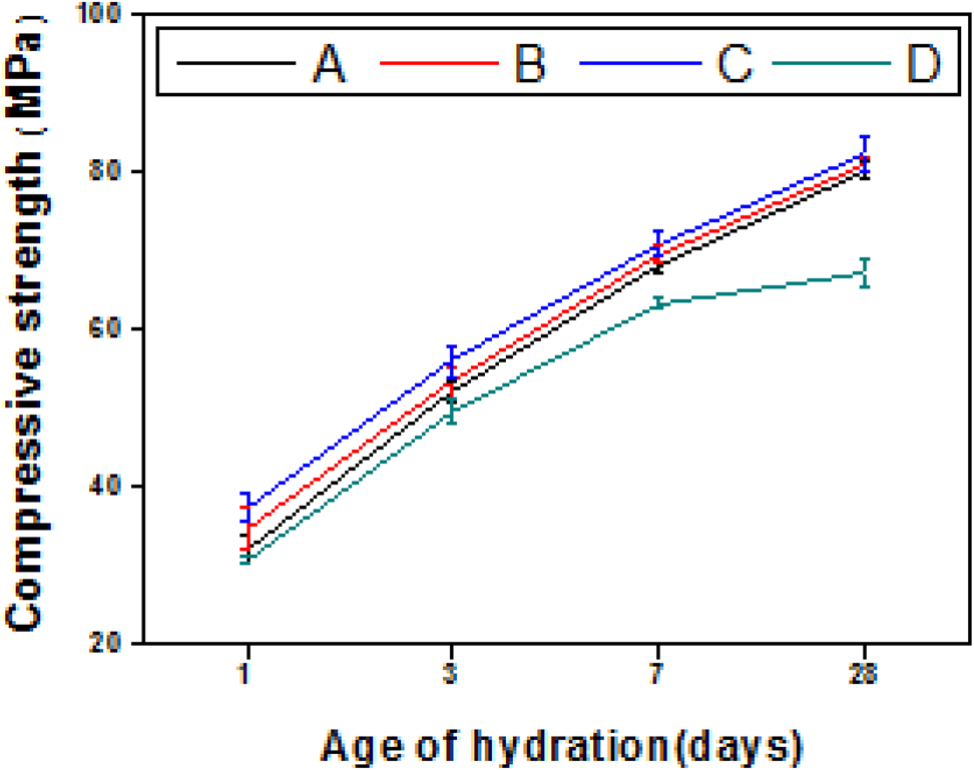
Compressive strength versus age of hydration for hardened blended cement pastes (mixes A–D).

The mechanical performance of the hardened pastes or concrete can be specified by performing the compressive strength test. The findings of this test can be concise as follows: (1) as a general trend all tested samples showed continuous strength rise with hydration progress (till 28 days), Fig. 6, this increase is ascribed to the progressive of the hydration reaction which induces the production of the diverse product that represents as a perfect binding center through the hardened matrix, the formed phases are: calcium silicate hydrates (C-S-Hs), calcium sulfoaluminate hydrates (AFm and AFt), (calcium aluminosilicate hydrates C-A-S-Hs as gehlenite and hydogarnet) and calcium aluminate hydrates (CAHs), these results will be supports by XRD, TGA/DTG, and SEM tests. (2) Upon replacing OPC with 5% or 10% RAS, by mass (mixes B and C; respectively) a slight improvement in the compressive strength values was recorded compared to paste made from mix A (control) along all testing ages. While a slight decline in the strength magnitudes was recorded when the replacement percentage of OPC with RAS reached 15%, Fig. 6. The notable improvement in the values of compressive strength in the case of replacing Portland cement with 5% or 10% RAS, by mass, could be correlated to the creation of extra hydrated yields; formed from the interaction between amorphous silica and alumina of sludge and lime generated from clinker hydration (pozzolanic reaction) forming an excess of CSHs, CAHs beside the creation of new phases namely as; MnCSH, CFH and CFSH^50,51^. Replacing portlandite which possesses weak hydraulic character with CSHs, CAHs which possess good hydraulic characteristics causes the noted strength improvement. Besides, these supplementary hydrates function as fillers for the existing pores in the hardened matrix and act as a perfect binding center between the remaining present unhydrated grains^37^. The notable observed downward in the values of strength of mix D (85% OPC–15% RAS) is mainly ascribed to the decline of clinker mineralogical phases within the composite which causes reduction of both the main products (CSHs and CAHs) produced from its hydration, besides reduction the produced quantity of portlandite that necessary for the pozzolanic reaction^49^. From the recorded strength values the optimal replacement of OPC by RAS is 10%, the same value was reported previously^52^.

**Figure 7:**
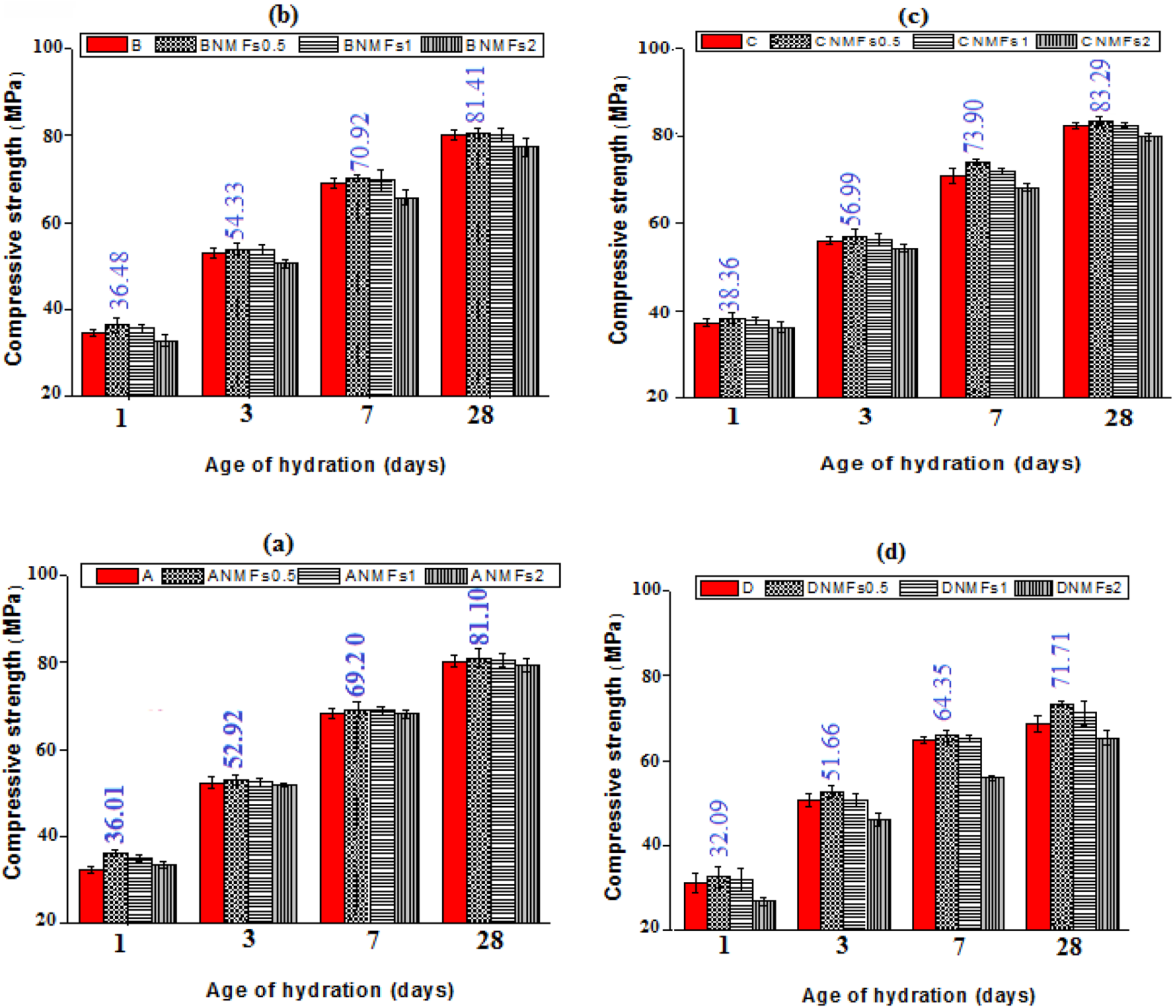
Compressive strength values of different hardened nanocomposites.

The impact of NMFs addition on the strength values of different composites was identified and clarified in Fig. 7a–d. Clearly, the inclusion of 0.5 and 1% NMFs in different pastes (mixes ANMFs0.5, ANMFs1, BNMFs0.5, BNMFs1, CNMFs0.5, CNMFs1, DNMFs0.5, and DNMFs1) induces a slight improvement in the strength values during all testing periods, while the inclusion of 2% NMFs induced slight decline in the values of compressive strength, Fig. 7a–d. Although the perfect nanoparticles dimension of the prepared NMFs (~ 13–40 nm), induced unexpected slight modification in the values of compressive strength, these results could be correlated to two factors; (1) the cubic geometric structure of NMFs particles (see Fig. 2) which hindered their pore filling and present weak points of contact between different phases present as reported in a previous study^45^. (2) it may be correlated to the poor dispersion of the nanoparticles especially when uses at high ratios^37,40,53^.

The slight decline in occurred in the values of compressive strength during almost all hydration intervals for ANMFs2, BNMFs2, CNMFs2, and DNMFs2 mixes (2% NMFs addition) compared to their references (mixes A–D) may be returned to the magnetic nature of NMFs particles. Manganese ferrite spinel is ferromagnetic in nature and its nanoparticles are interacting magneto statically, therefore, they attract each other which induced their bad dispersion and promoted their coalescence^46^.

In conclusion, the findings assured that 90% OPC–10% RAS–0.5% NMFs composite (mix CNMFs0.5) could be reported as the optimal choice for general construction applications as it displayed the best mechanical behavior among all tested nano-blends. These results will be supported by further performed tests.

### Bulk density (BD)

**Figure 8:**
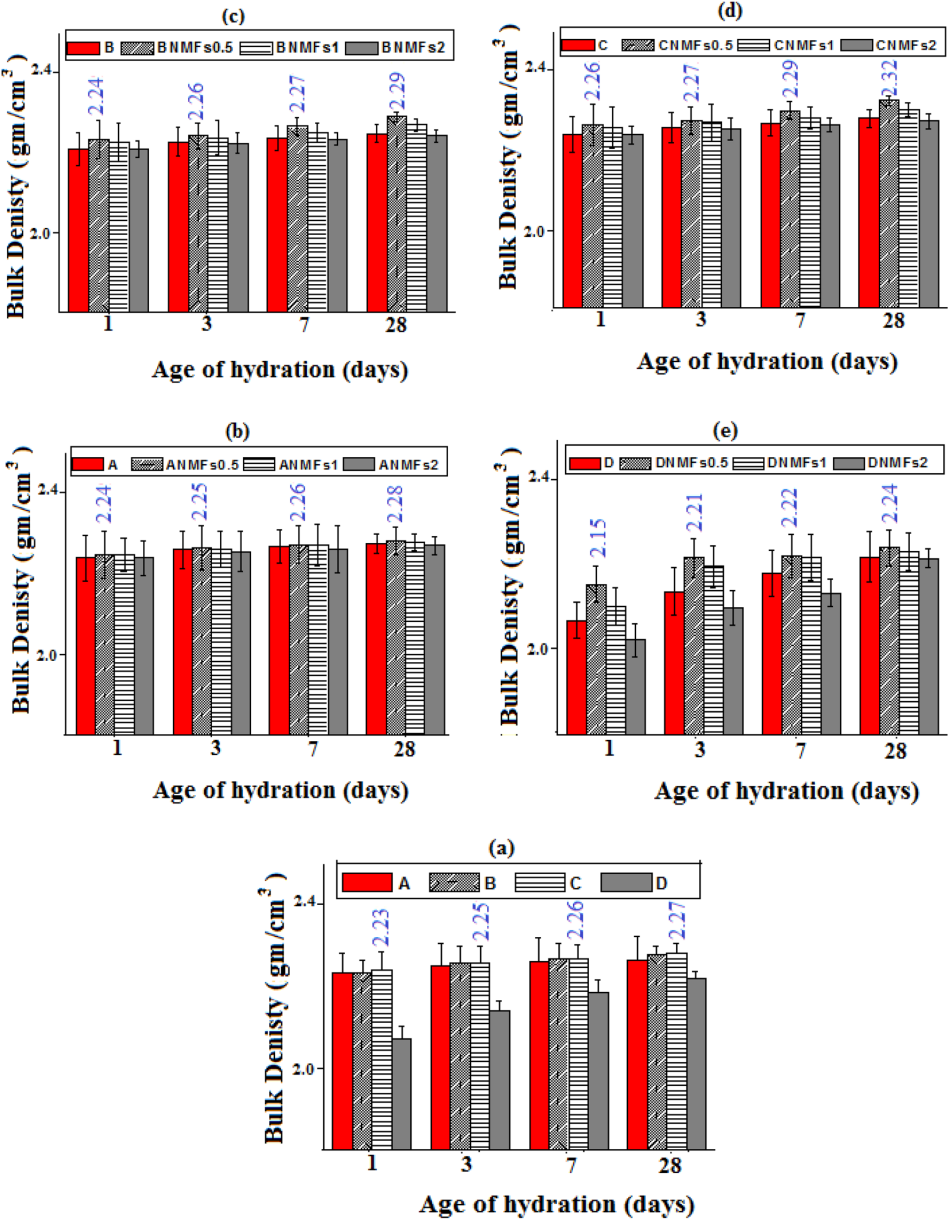
Bulk density values of different hardened composites.

The densification progress of different composites with the progress of the hydration process was followed by measuring the BD values (g cm^−3^). The BD values for different blends indicated a continuous gradual rising with hydration ages, Fig. 8a–e. These findings are assigned to the progress of the hydration process and the accumulation of different hydrated phases within the available pores along the hardened matrix, producing compact and dense structures^48^. Figure 8a indicates that specimens made from mixes B and D (using 5% or 10% RAS, by mass) presented a notably higher BD value (at all curing times) relative to a blank (mix A). This finding is correlated to the formation and accumulation of diverse types of hydrated phases that formed via the pozzolanic interaction between available liberated calcium hydroxide and RAS, creating a denser matrix with high strength^4^. On contrary, the BD values when using 15% RAS (mix D) are lower and/or comparable to a blank. These findings are consistent with the previously mentioned compressive strength data and ascribed to the formation of lower quantities of different hydration products owing to the dilution effect of OPC phases by 15%^34^.

The inclusions of different doses of NMFs (30.73 m^2^ g^−1^ and ~ 16 nm) up to 1% improve the BD of all composites during almost all intervals of hydration, Fig. 8b–e. This enhancement could be correlated to the nano-filling effect and the acceleration effect of NMFs particles for the hydration process via acting as active sites. The net result of the inclusion NMFs parties is the generation of additional quantities of yields like CSHs, CASH, CAHs, CFH, CFSH, and MnCSH that accumulated inside the available pores within the hardened composite matrix giving a more compact and dense structure^34,45,51^. Besides, the inclusion of 2% of NMFs induces a slight decline in the BD values, due to the agglomeration effect of NMFs particle as mentioned previously^4^. All these results agreed with the compressive strength test results and will receive further support via the XRD, TGA/DTG, and SEM techniques. The BD results revealed that mix CNMFs 0.5 presented the highest BD values matched to those of other mixes. This result agreed and confirmed the obtained compressive strength results.

### Total porosity

**Figure 9:**
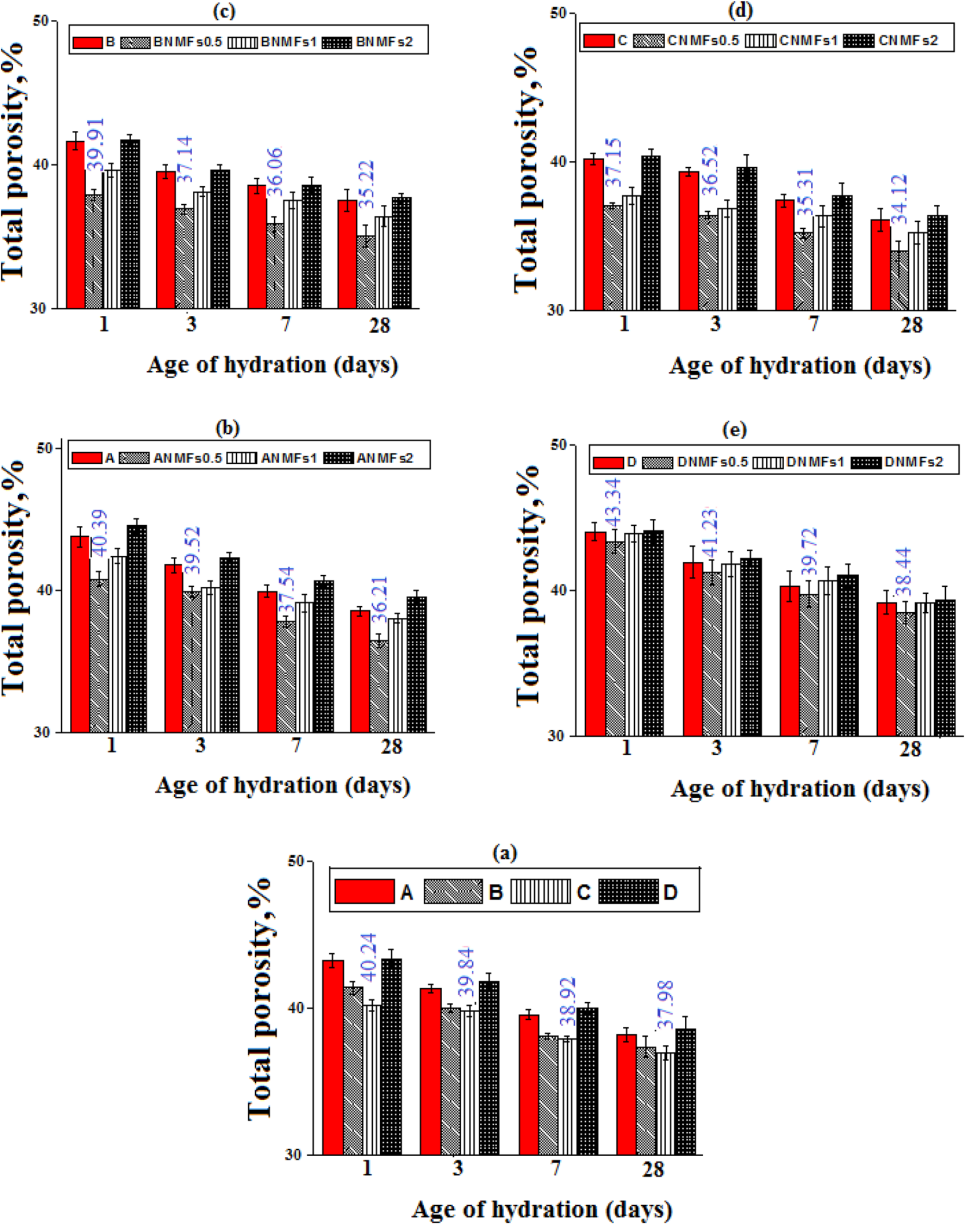
Total porosity values of different hardened composites.

Figure 9a displays the percentage values of total porosity (TP%) of different composites after different testing periods. As a general trend, the TP% decreases for tested composites showed continuous reduction hydration till 28 days of curing. Generally, specimens of mix B and mix C indicated lower TP (%), while mix D specimens showed relatively higher values relative to mix A. Moreover, the insertion of 0.5 and 1% of NMFs induced lower TP% for the hardened nanocomposites, Fig. 9b–e. The addition of 2% of NMFs to different blends gives hardened pastes with comparable and/or higher relative TP% values, compared to their controls (mixes A–D). As the BD values increase, the TP% decrease, and the compressive strength values increase^48^. Obviously, the obtained TP% values are agreed with the obtained results of the strength and BD test and are ascribed to the same reasons mentioned previously. Figure 9c displays that, CNMFs0.5 composites present the lowest TP%. Also, these results will receive further support from XRD, TGA/DTG, and SEM analysis.

### Water absorption (WA %)

**Figure 10:**
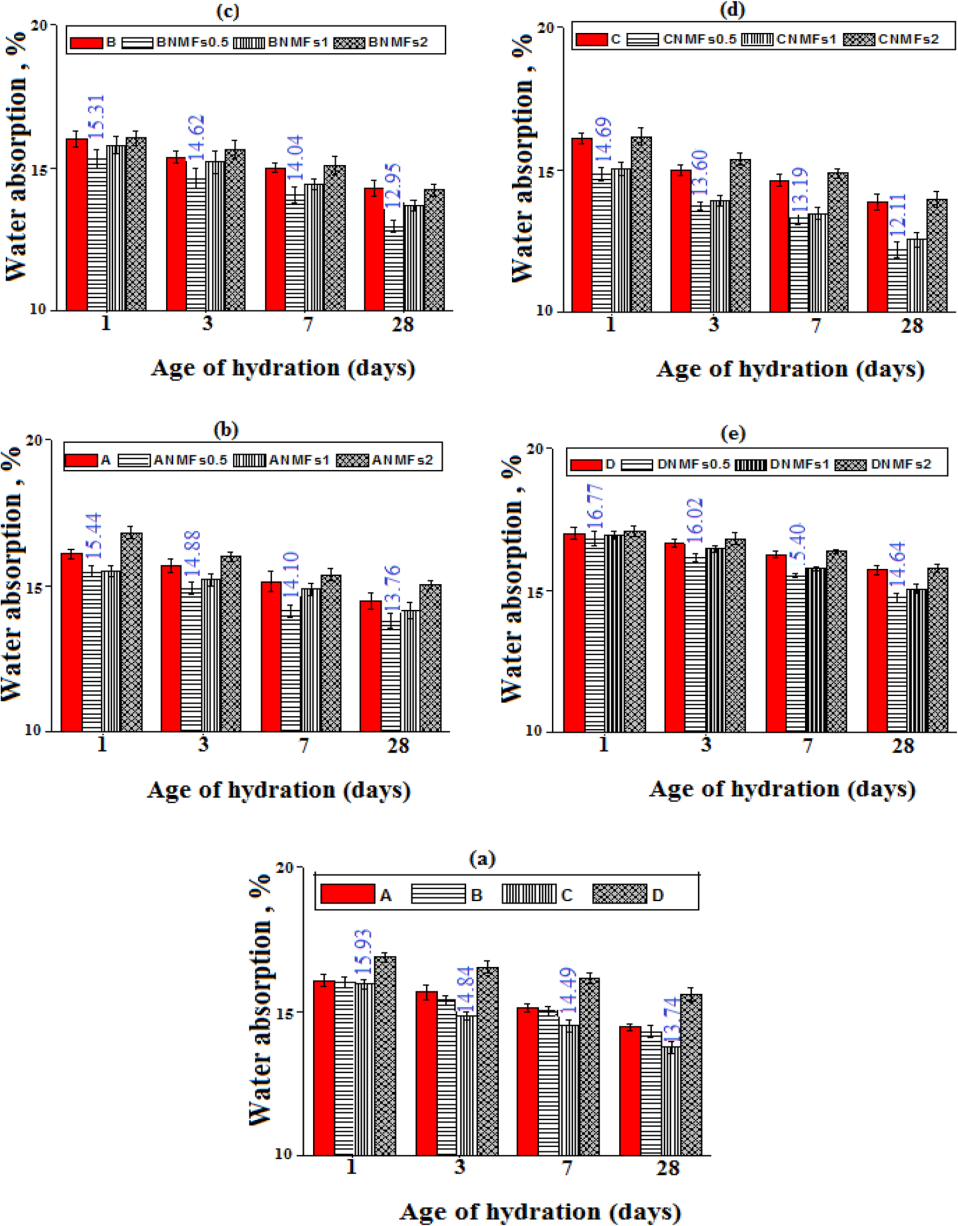
Water absorption values of different hardened cement composites.

The degree of porosity of the hardened specimens can be identified via the determination of the WA%^2^. The percentage values of WA% for various tested mixes were measured and displayed in Fig. 10a–e. As demonstrated from the obtained WA% values, indicated the same trend as TP% values for all tested mixes. Obviously, as the B.D values increase the TP% and the WA% decreases^4^. Clearly, all the obtained results for pore structure analysis (BD.TP, and WA) are correlated with each other and agreed with the compressive strength test results.

### Stability against firing (SAF)

**Figure 11:**
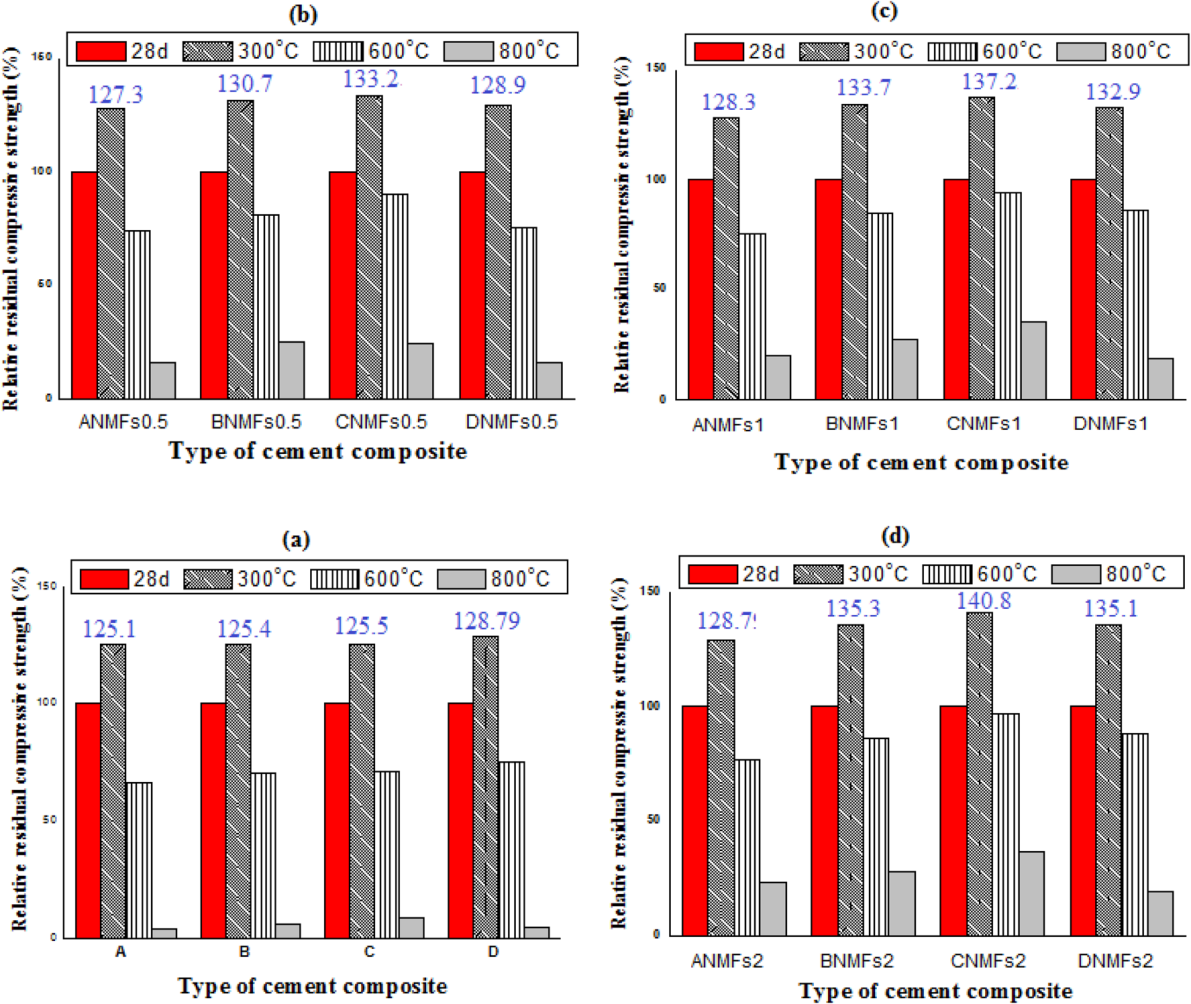
Relative residual compressive strength values for different fired cement composites after slow cooling in air.

The stability of different hardened blends against firing at 300, 600, and 800 °C for 3 h and cooled slowly in the air was identified and represented as the percentage of residual compressive strength (RS%) in Fig. 11a–d. The findings of this test are: (1) all composites indicated a sharp increase in the RS (%) strength values when heating at 300 °C and cooled in air, compared to their recorded values at 28 days hydration, after that a significant diminishing in residual strength upon firing at 600 or 800 °C was recorded, (2) inclusion of different amounts of NMFS in neat cement and/or blends boosts the SAF at different temperatures. The sharp boosting in residual strength (%) values for gradually cooled samples after firing at 300 °C is ascribed to the self-autoclaving (hydrothermal reaction) generated between the evaporation of physically adsorbed H_2_O and remaining unreacted clinker phases, this internal autoclaving generates extra hydration product that fills the available pores as well as present additional binding centers between different constituents present in the hardened composites^2,48^. The observed diminish of RS (%) strength in gradually cooled specimens after being heated at 600 or 800 °C is correlated to the deterioration of most hydration products (CSHI, CSHII, CASHs, CAHs, CFSH, CFH, MnCSH, and CH) and formation of microcracks along the matrix which has a strong negative impact on composites strength^54^. All nanocomposite pastes (incorporating diverse doses of NMFs) displayed higher resistance for firing (high RS%) at all applied temperatures and cooling in the air relative to their controls after 28 days of hardening, also the SAF for these nanocomposites increases with NMFs percent. The positive impact of NMFs on the SAF of different composites is correlated to its filling effect on the present pores, its acceleration effect on the internal autoclaving reaction by acting as a nucleating center for product formation^45^, and the magnetic nature of ferromagnetic spinel nanoparticles that leads to magnetostatic interactions between the particles, promoting the production of harden matrix possess high resistance to fire deterioration^46^.

**Figure 12:**
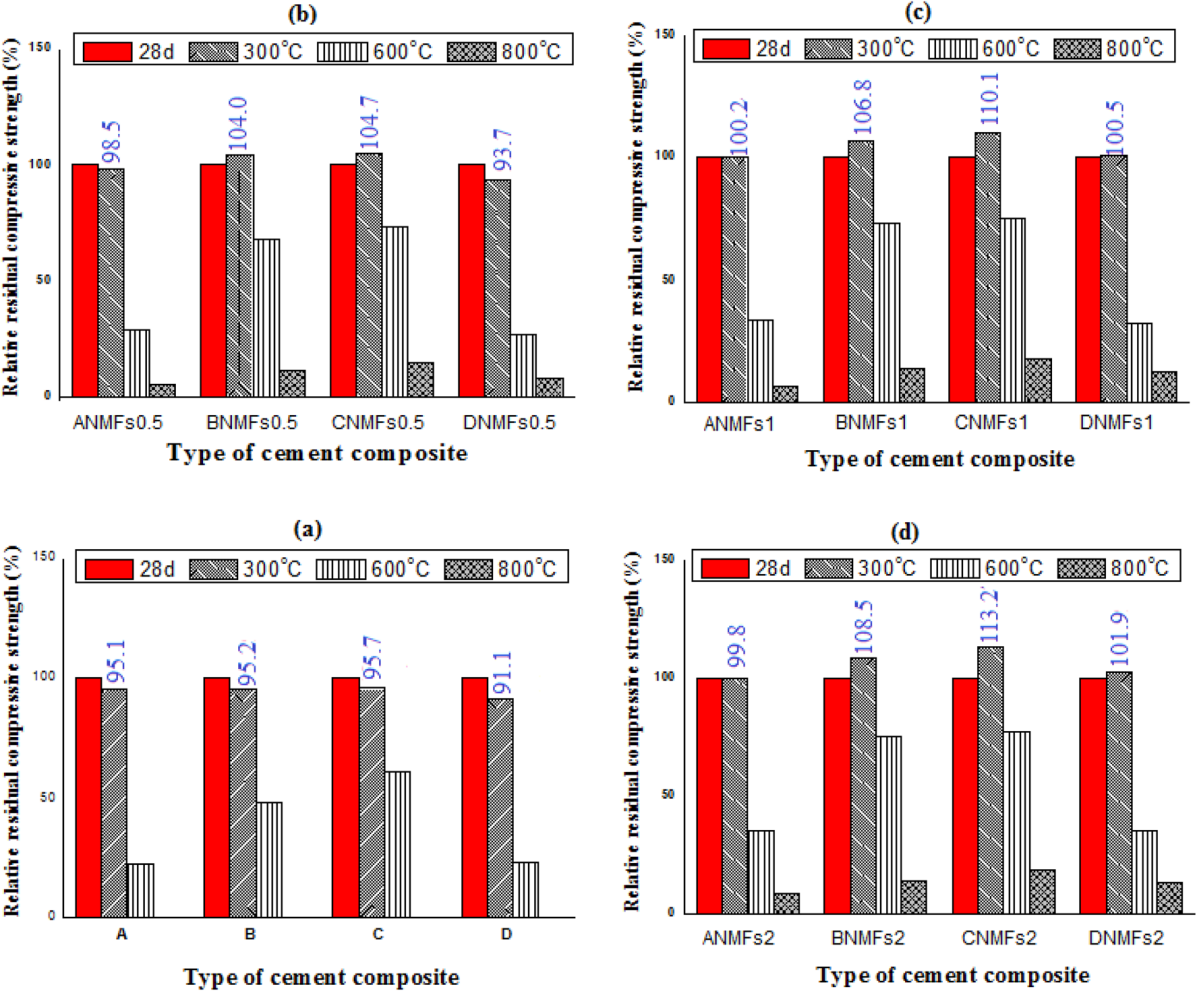
Relative residual compressive strength values for different fired cement composites after suddenly cooled.

Figure 12a–d. display the RS (%) values for different composites after rapid cooling (cooling in water) after firing at 300, 600, and 800 °C for 180 min. All composites undergo a continuous loss in strength upon being cooled in water after heating at different reported temperatures (300–800 °C), and the degree of loss in strength increases with temperature, Fig. 12a–d. Clearly, the degree of depletion in strength is higher (RS% lower) for all composites as compared to their loss when undergoing slow cooling after treatment at the same temperature. The intensive reduction in compressive strength for the rapidly cooled samples values is ascribed to the thermal shock to occur by the sudden cooling of fired specimens that causes the creation of several cracks as well as enlarges of the microcracks present along the composite matrix^34^. As previously mentioned in many studies using nanofiber/tubes as nano montmorillonite (NM) and carbon nanotubes (CNTs), Titania nanoparticles (TN)^53–55^ may delay the microcrack propagation by boosting tensile and flexural strength and improving the microstructure homogeneity in the cement matrix^53,56–58^.

The percentage relative compressive strength (RCS) (relative to their CS after 28 days) for all fired specimens after slow and rapid cooling are clarified in Figs. 11 and 12, respectively. The RCS percentage values after firing at 300 °C and cooled slowly are 125.16, 125.4, 125.53, and 128.79 for (Mixes A–D).

The RCS percentages (relative to control at 300 °C and cooled slowly) for (Mixes ANMFs0.5-ANMFs2) are 127.37, 128.32, and 128.79%, respectively, 130.74, 133.78, and 135.39 for (Mixes BNMFs0.5-BNMFs2); respectively, 133.26, 137.23, and 140.87 for (Mixes CNMFs0.5-CNMFs2); respectively, and 128.90, 132.94, and 135.16, for (Mixes DNMFs0.5-DNMFs2); respectively, Fig. 11a–d. Visibly, the obtained SAF results recommend the nanocomposite contains 90% OPC–10% RAS–2% NMFs to be the best selection for thermal application as it presents the highest % RS (highest SAF) at both gradual and sudden cooling at all reported temperatures, the recorded RS% values for this composite are 133.7, 137.2, and 140.8 for slowly cooled specimens fired at 300, 600, and 800 °C; respectively. While the recorded RS % values for this composite are 104.7, 110.1, and 113.2 for rapidly cooled specimens fired at 300, 600, and 800 °C; respectively. These findings nominated this composite to be the perfect selection for fire resistance application.

### Phases development

#### X-ray diffraction analysis (XRD)

**Figure 13:**
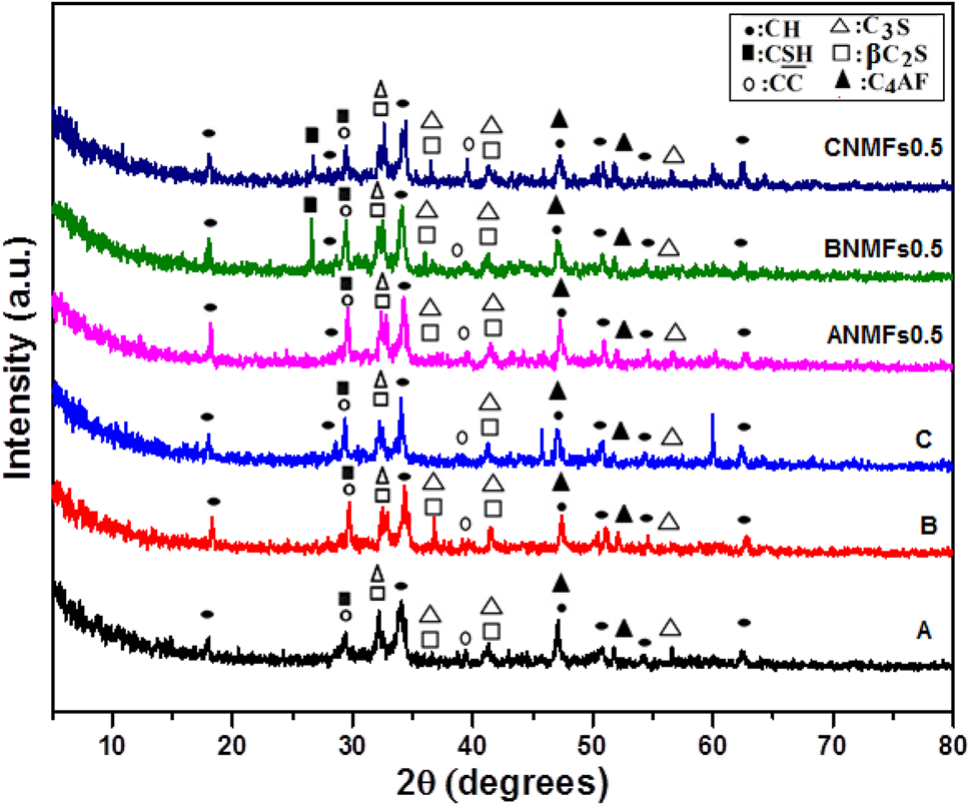
XRD patterns of hardened composites made from mixes (**a**–**c**), and nanocomposites made from mixes (ANMFs0.5–CNMFs0.5) at 7 days of hydration.

**Figure 14:**
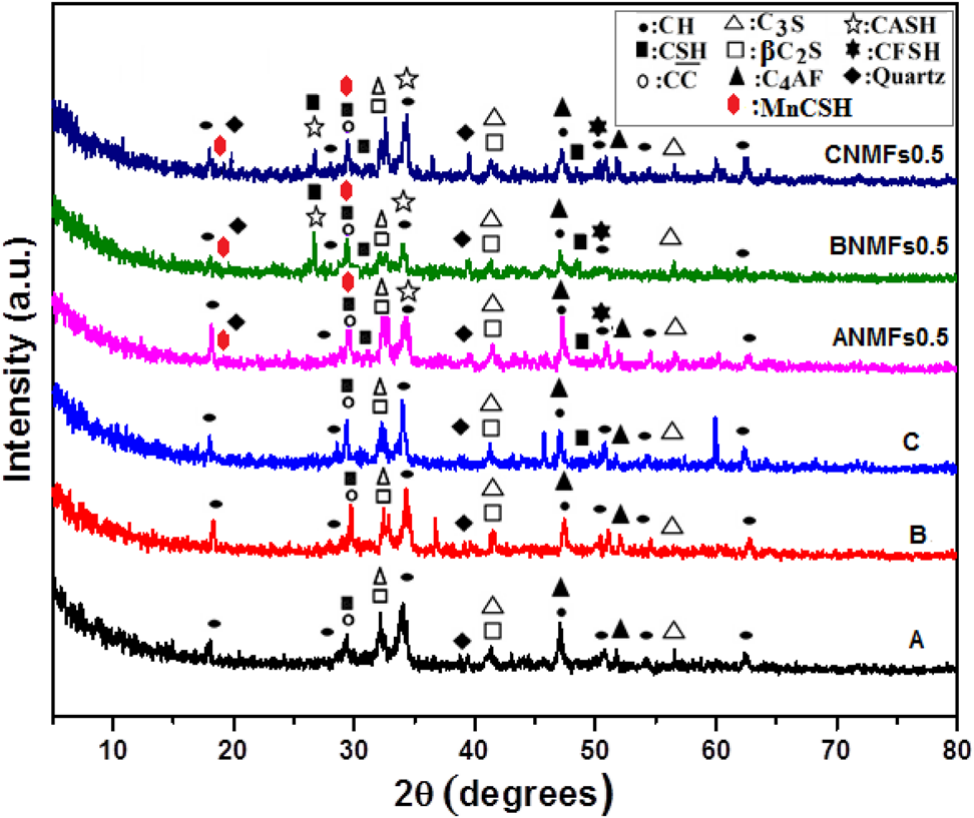
XRD patterns of hardened composites made from mixes (A–C), and nanocomposites made from mixes (ANMFs0.5–CNMFs0.5) at 28 days of hydration.

Figures 13 and 14 show the phases of development for composites composed of 100 OPC, 95 OPC-5 RAS, 90 OPC-10 RAS, OPC-0.5 NMFs, 95 OPC-5 RAS–0.5NMFs, and 90 OPC–10 RAS–0.5NMFs at 7 and 28 days hydration. The characteristics peaks of CSH and Ca (OH) phases were identified in XRD patterns for these mixes, besides the peaks that characteristics to the unreacted quartz (crystalline silica), C_3_S (3CaO.SiO_2_), and β-C_2_S (2CaO. SiO_2_). Calcium carbonate was observed at 2θ of ~ 29.32° formed from the reaction of lime (CH) with atmospheric CO_2_ gas^52^. The XRD patterns of nanocomposites (composites admixed by 0.5 NMFs) revealed the appearance of new phases especially at 28 days' hydration namely: ilvaite (CFSH) which is located at 2θ 51°, MnCSH at 2θ 12.84, 18.36 and 29.6°, Nchwaningite [Mn_2_ SiO_3_(OH)_2_ H_2_O] (PDF#83-1006), Rancieite [(Mn, Ca) Mn_4_O_9_.3H_2_O] (PDF#058-0466), Glaucochroite [(Ca, Mn)_2_SiO_4_ (PDF# 14-376) located at 2θ 16.9°, and CFH^51^. These results support the obtained test results of compressive strength, BD, TP%, and WA% reported previously. The formation and later accumulation of these supplementary products promoted the development of densify microstructure possessing good mechanical characteristics and durability.

#### Differential thermogravimetric analysis

**Figure 15:**
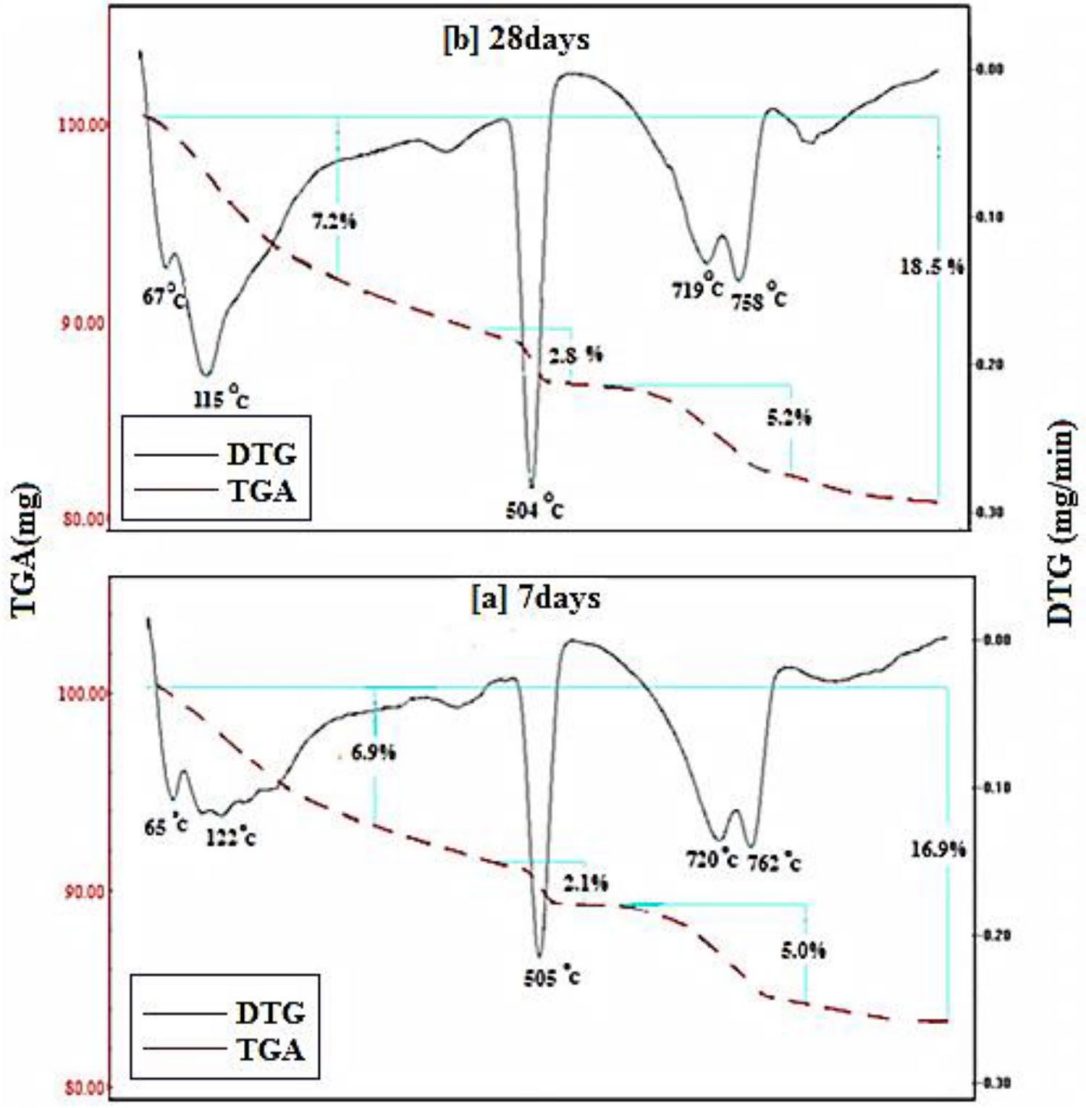
TGA/DTG thermograms of hardened specimens made from mix A at (**a**) 7 days and (**b**) 28 days hydration.

**Figure 16:**
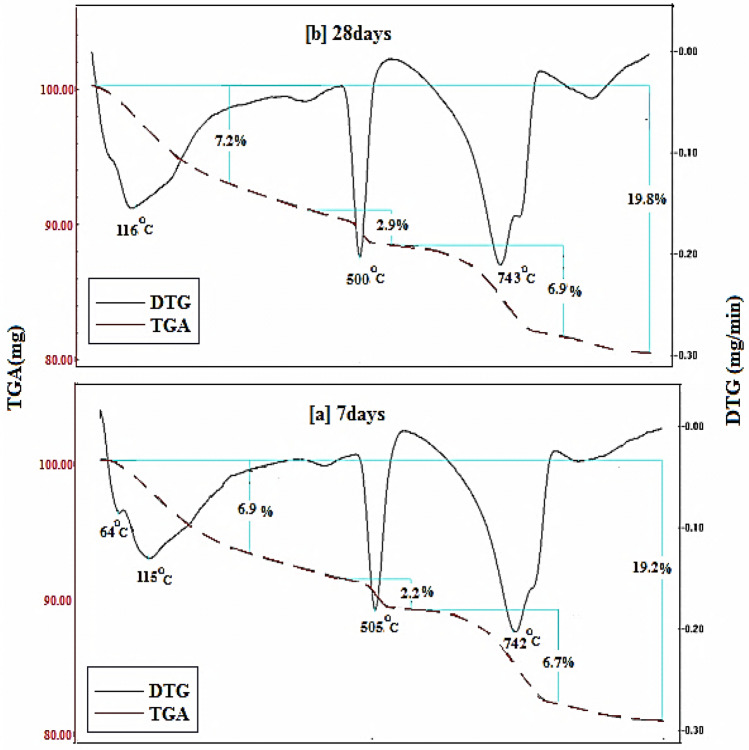
TGA/DTG thermograms of hardened specimens made from mix ANMFs0.5 at (**a**) 7 days and (**b**) 28 days hydration.

**Figure 17:**
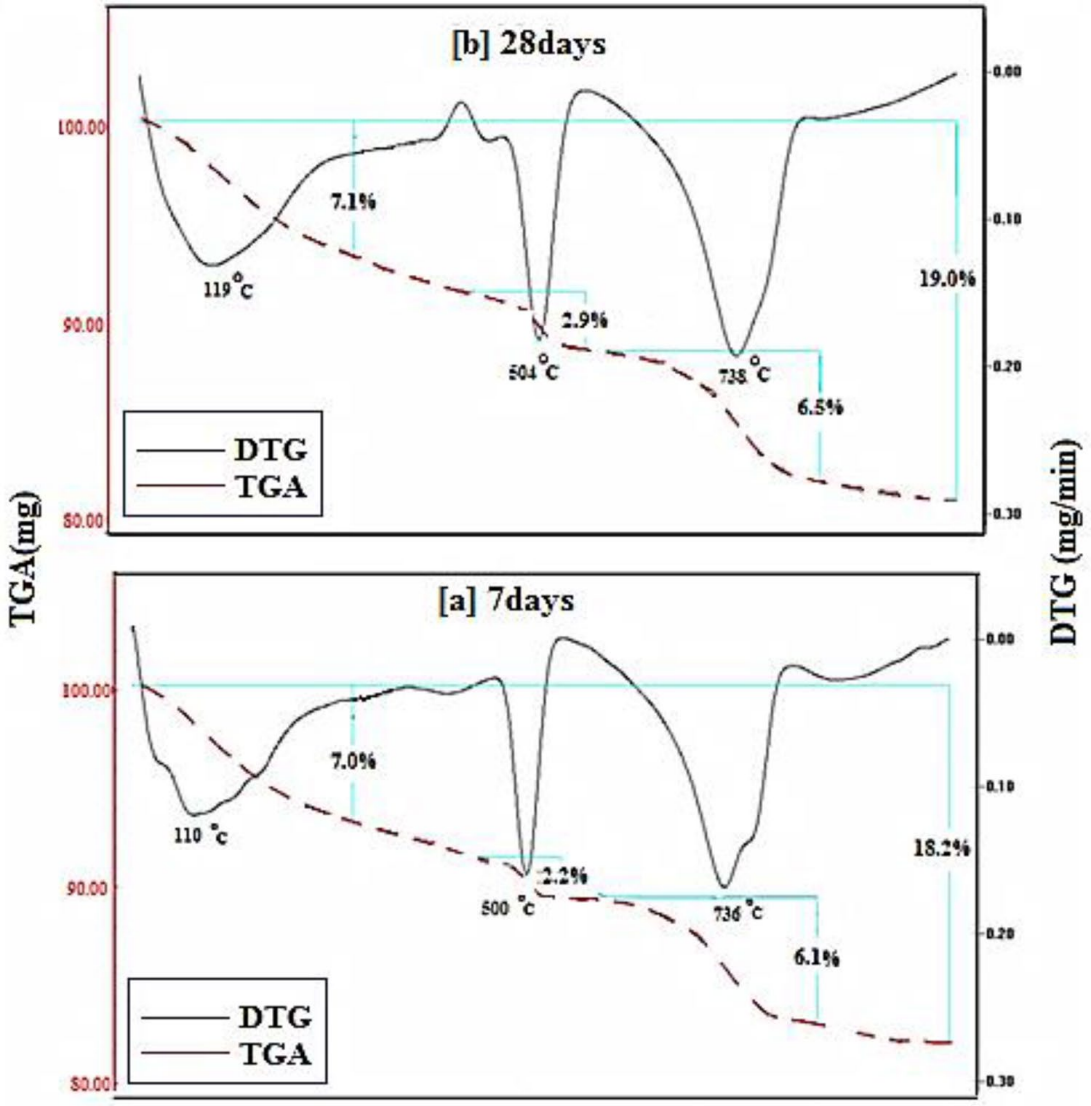
TGA/DTG thermograms of hardened specimens made from mix C (**a**) 7 days and (**b**) 28 days hydration.

**Figure 18:**
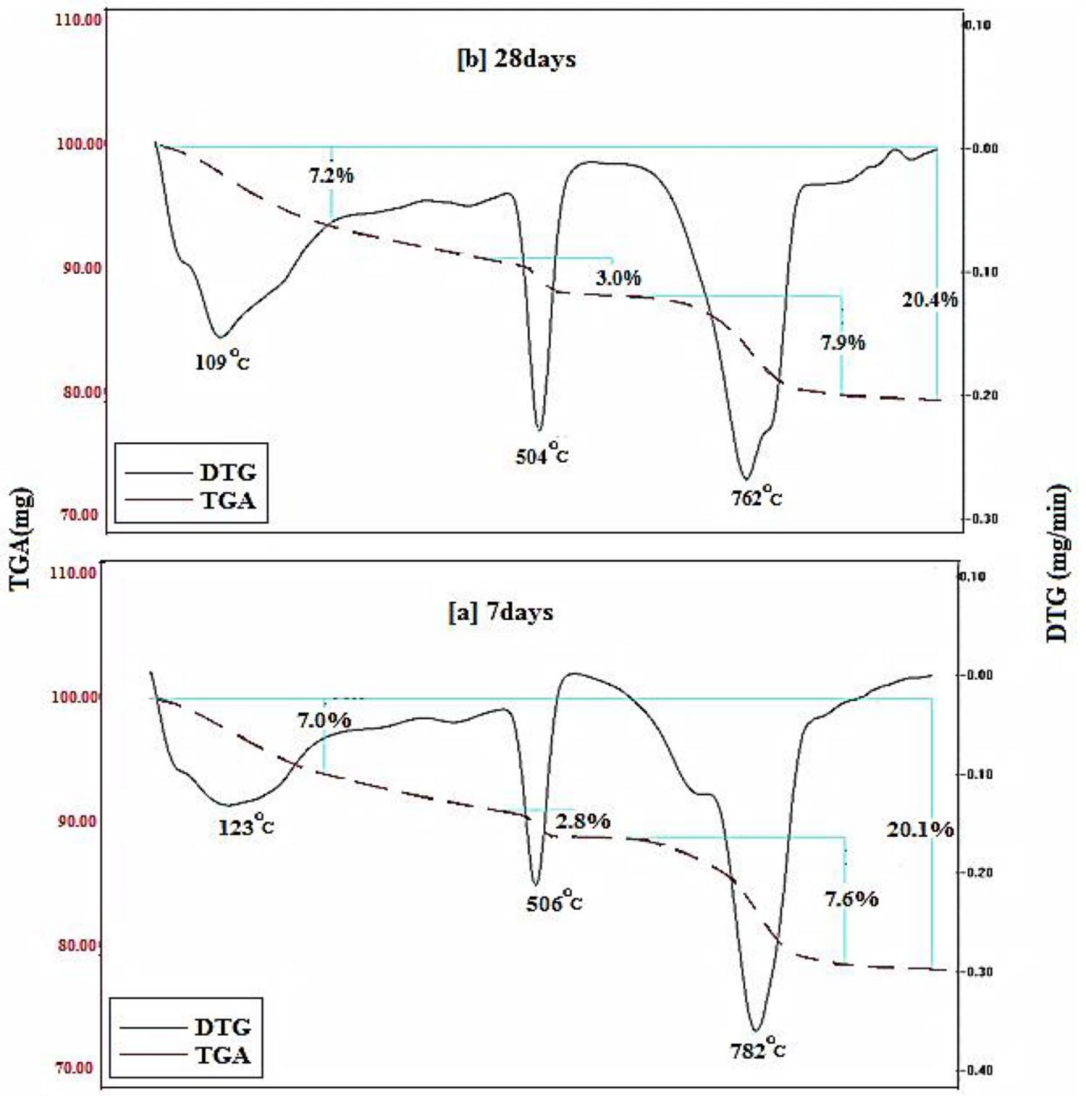
TGA/DTG thermograms of hardened specimens made from mix CNMFs0.5 at (**a**) 7 days and (**b**) 28 days hydration.

Figures 15, 16, 17, 18 demonstrates the TGA/DTA data for OPC, OPC-0.5NMFs, 90 OPC-10 RAS, and 90 OPC-10 RAS-0.5 NMFs (Mixes A, ANMFs 0.5, C, and CNMFs 0.5) at 7 and 28 days of hydration. The TGA/DTG results display three endothermic peaks located at ~ 70–180 °C, ~ 505 °C, and a double peak at 720 and 780 °C, for neat OPC samples at the selected times (7 and 28 days). The peak implanted at ~ 70–180 °C is devoted to the deportation of uncombined water and disintegration of the amorphous part of CSHs gel and calcium aluminate hydrates (CAHs), Fig. 15a,b^4^. The mass loss of this peak after 3 and 7 days of hydration is 6.9%, and 7.2%, respectively. The endotherm that appeared at 505 °C is correlated to the dehydration of CH (portlandite)^4,59^. The mass loss of this peak is 2.1% and 2.8% after 3 and 28 days of hydration, respectively. The observed increases in % mass losses in both endotherms are correlated to the formation of extra amounts of hydration phases (CSHs, ettringite, AFm, and CH) with the progress of the hydration process from 7 to 28 days^2^. The double peak present at 720 and 780 °C is ascribed to the degradation of calcium carbonates with different crystallinity, because of the carbonation of the specimens during its handling. The% mass loss values of these peaks are varied according to the degree of crystallinity and the degree of carbonation^51,59^.

Figure 16a,b displays the TGA/DTG result of OPC-0.5 NMFs nanocomposite (mix ANMFs 0.5) at 7 and 28 days of hardening. The TGA/DTG behavior of this composite is identical to neat OPC (mix A) but with a higher % total mass loss value. The mass loss % of the diverse hydration yields is 16.9 to 18.5% (for OPC) and 19.2, and 19.8 (for OPC-0.5%NMFs) at 7 and 28 days; respectively. This finding ascertains the affirmative impact of NMFs on the acceleration of the hardening reaction of OPC and the simulation of the production of new phases (MnCSH, MnSH, and CFSH) as demonstrated from XRD data. This intern promoted the creation of hardened composite having good mechanical properties, higher BD, lower TP%, and lower WA values as previously mentioned in this study.

Figure 17a,b represents the TGA/DTG thermograms for hardened specimens that possess 90 OPC-10 RAS (mix C). Obviously, similar endotherms and behavior as blank are observed. The % mass loss of the peak present at ~ 70–180 °C is 7.0 and 7.1 while the peak present at 505 °C is 2.2 to 2.9 at 7 and 28 days; respectively. The variation in these values is ascribed to both the degree of crystallinity and quantity of the formed products. The detected peak at (~ 740 °C) is referred to as carbonates decomposition.

Obviously, the overall % mass losses for the formed hydrates are 18.2 and 19 at 7 and 28 days: respectively. The notable raising in the total mass loss relative to a blank (16.9 and 18.5) is related to the generation of extra amounts of nearly amorphous and crystalline CSH phase from the pozzolanic interaction between CH liberated from OPC hydration, and silica and alumina phases of RAS waste. These outcomes are compatible with the physical and mechanical test results reported mentioned previously.

The TGA/DTA results for 90 OPC-10 RAS-0.5 NMFs nanocomposite (mix CNMFs0.5) after 7 and 28 days hardening are identified. The TGA/DTG findings of this mix demonstrated similar peaks and behavior as mix C, Fig. 18a,b. The overall mass reduction percentages are 20.1 and 20.4% at 7 and 28 days, respectively; these percentages are relatively higher than that of other mixes (A, ANMFs0.5, and C). The notable higher overall mass loss of these nanocomposites is credited to the reinforcing effect of NMFs particles as discussed previously. Also, the TGA/DTG results are corroborated with all previously mentioned results (RS, TP, BD, and WA).

### Microstructure

**Figure 19:**
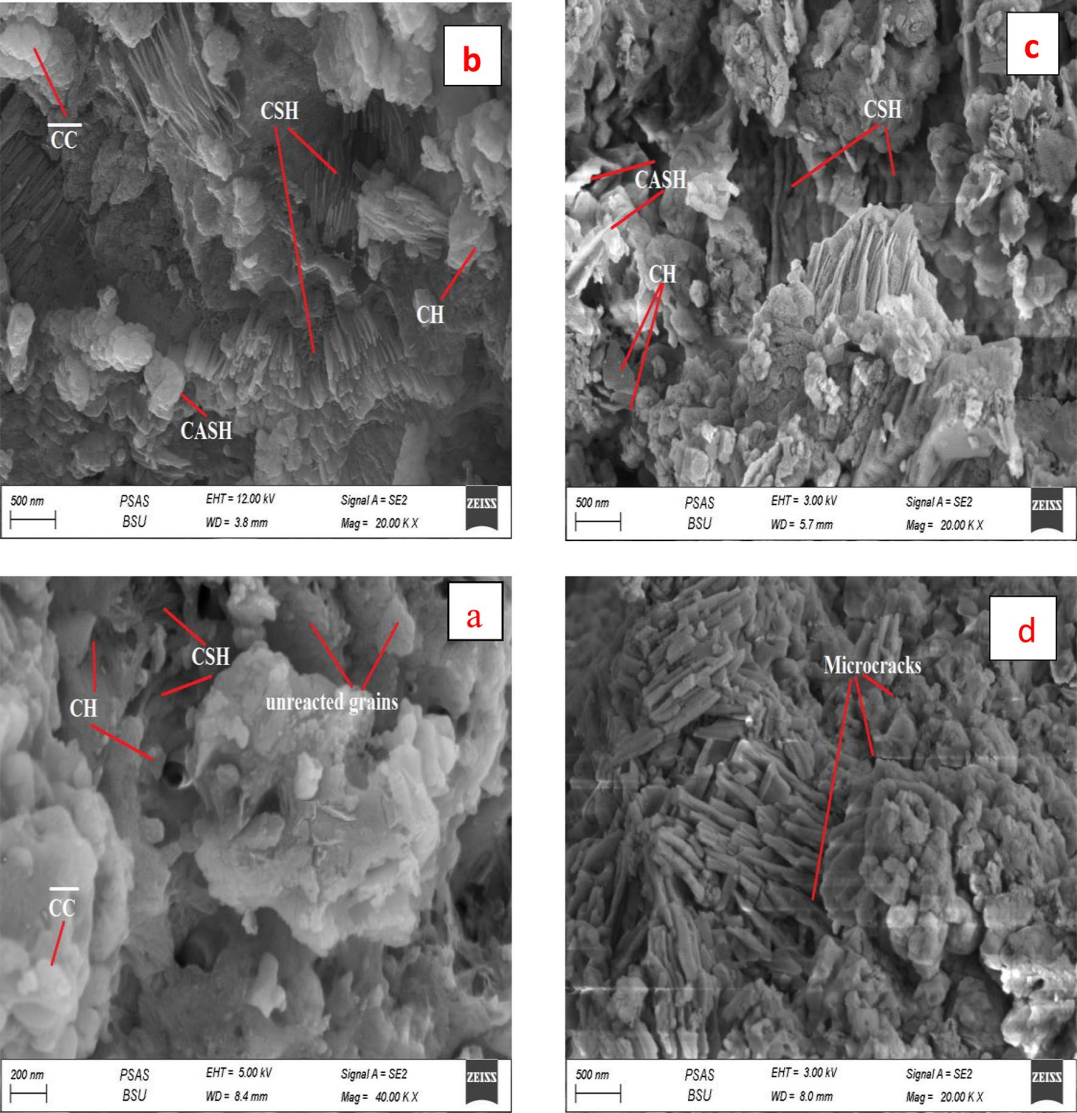
SEM micrographs of hardened specimens made from mix A: (**a**) after 7 days, (**b**) after 28 days, (**c**) after firing at 300 °C and (**d**) after firing at 800 °C.

Figure 19a,b clarifies the morphological features for mix A specimen at 7 and 28 days hydration intervals. The SEM investigation of this mix affirms the appearance of a porous matrix diffused by some fibrous and ill crystals of CSH as a principal product from clinker hydration at 7 days. Also, small hexagonal plates of CH were detected along the matrix, little amount of CC̅ besides a large quantity of unreacted clinker grains could be well distinguished, Fig. 19a. The micrographs of this mix at 28 days' hydration displayed a dense matrix composed of excessive quantities of microcrystalline and fibrous CSH, the large amount of CH also existed as stacked hexagonal crystals. Besides, little crystals of CC̅ are found in the matrix, Fig. 19b. The SEM micrographs also illustrated the existence of a few voids in the matrix accessible for the deposition of new hydration products.

The effect of firing on the microstructure of neat OPC samples is investigated and given in Fig. 19c,d. Firing the hardened OPC sample at 300 °C and cooling it in the air (slow) induced the formation of a highly compact matrix composed of an excessive amount of nearly amorphous fibrous C–S–H (the major hydration products) interlocked by the hexagonal plates of CH. These microstructure features reflect the internal autoclaving reaction of unreacted OPC grains that causes the generation of an extra product (CSH, CH, CAHs, CASHs), promoting the formation of the dense structures having advanced properties (high strength, low porosity, high density). This finding confirmed the obtained data from RS, BD, WA, and TP tests. The microstructure of the OPC specimen exposed to a higher temperature (800 °C) and gradually cooled, revealed the formation of several micro-cracks, with the absence of any hydration products because of their decomposition. Certainly, the degradation of hydration products and the existence of microcracks are essential reasons for the deterioration of these specimens, Fig. 19d.

**Figure 20:**
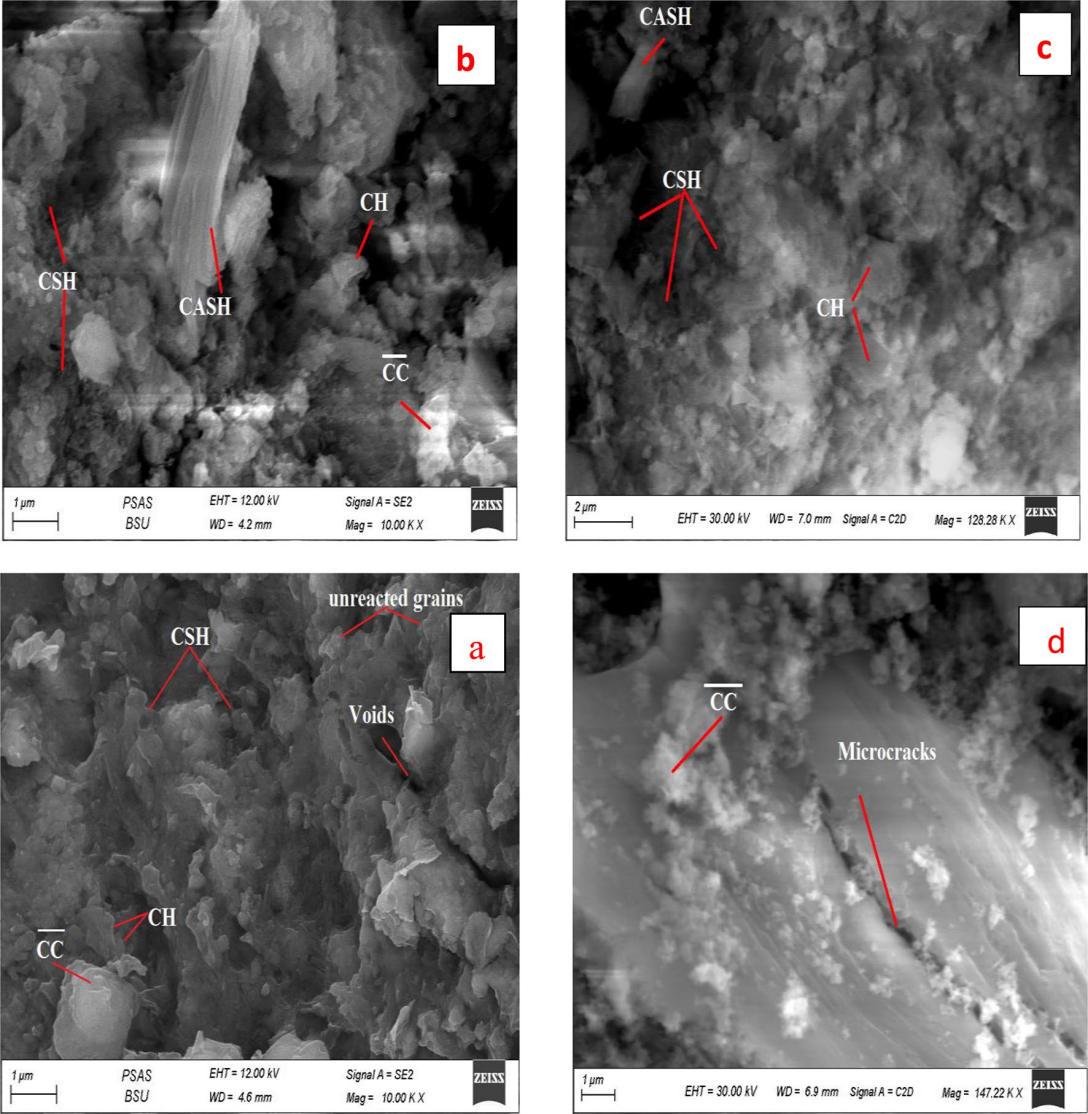
SEM micrographs hardened nanocomposite made from mix CNMFs0.5: (**a**) after 7 days, (**b**) after 28 days, (**c**) after firing at 300 °C and (**d**) after firing at 800 °C.

Figure 20a,b displays the microstructure of hardened specimens made from 90% OPC + 10% RAS + 0.5% NMFs (mix CNMFs0.5) at 7 and 28 days, respectively. The SEM micrographs of these nanocomposites indicated the following: (1) specimens hydrated for 7 days showed less dense microstructure has little amount of hydrated phases, and excess particles of unreacted clinker compared to the microstructure of specimens hydrated for 28 days, (2) Replacing cement with 10% RAS, induce the production of excess amounts of hydrated phases (CSH, CAHs, CASHs), this intern enhance the microstructure features and the densification characteristics of these nanocomposites compared to the neat OPC paste at the same curing ages, Fig. 20a,b and (3) the activation rule of NMFs particle was ascertained by the dense and nonporous matrix displayed by the SEM images of these nanocomposites (Fig. 20a,b). The NMFs particles induce the formation of enormous hydrated phases as its rule as active site and via its reaction and formation of a diverse product that appeared in the micrographs like; micro rods and fibrous crystals of CSHs, plates of CASHs, fine crystals of CFSH, CFH, MnCSH and fibers of AFt (3CaO·Al_2_O_3_·3CaSO_4_·32H_2_O); all these products are interlocked with each other and intermixed with little hexagonal crystals of CH, producing compact, non-porous structure having good mechanical properties^60^. The SEM study fastened the physico-mechanical results obtained in this study.

The effect of firing at 300 and 800 °C and cooling in the air on the microstructure of these composite is displayed in the SEM image, Fig. 20c,d. The specimens made from 90 OPC-10RAS-0.5NMFs indicated stable microstructure having good thermal resistance compared to control specimens (neat OPC) when exposed to the same conditions. The inclusion of NMFs particles in this composite promotes its resistance to thermal degradation as a result of its magnetic nature^47^. The microstructure upturn of this blend has also correlated with the presence of RAS as previously discussed.

## Conclusions

The impact of the inclusion of MnFe_2_O_4_ spinel nanoparticles on the physical, mechanical, microstructure, and deterioration characteristics of blended composites prepared from OPC, and RAS is reported in this article.

The outcomes of this investigation can be briefed as follows:

1. The obtained results fastened the suitability of utilization of RAS waste for replacing OPC (up to 10%) to prepare construction material having enhanced mechanical characteristics and durability.
2. The inclusion of different doses of MnFe_2_O_4_ spinel nanoparticles within the OPC-RAS pastes, motivates the formation of hardened nanocomposites with improved physico-mechanical performance and stability against firing.
3. The outcomes of different tests affirmed that 90% OPC–10% RAS–0.5% NMFs composite could be considered the best choice for general construction applications as it displayed the highest compressive strength values and highest RS% after firing among all other tested nanocomposites.
4. From the economic point of view composite replacing OPC by 10% RAS share in reducing waste disposal costs (landfill tax), offering an alternative use for recycled water-treated plant sludge, without prejudging on either cost or quality.
5. TGA/DTG, XRD, and SEM techniques affirmed the activity of NMFs particles, as they demonstrated the presence of enormous types of hydration products (such as MH, CAHs, MnCSH, CFSH, CSHs, AFt, AFm, CASHs, and CFH). These products boosted the mechanical and degradation resistance of the nanocomposites upon firing.

## Data Availability

All data generated or analyzed during this study are available upon request from all authors of this paper.
